# A Leptin Receptor Antagonist Attenuates Adipose Tissue Browning and Muscle Wasting in Infantile Nephropathic Cystinosis-Associated Cachexia

**DOI:** 10.3390/cells10081954

**Published:** 2021-07-31

**Authors:** Alex Gonzalez, Wai W. Cheung, Elliot A. Perens, Eduardo A. Oliveira, Arieh Gertler, Robert H. Mak

**Affiliations:** 1 Division of Pediatric Nephrology, Rady Children’s Hospital, University of California, San Diego, CA 92093-0831, USA; alg022@health.ucsd.edu (A.G.); w5cheung@health.ucsd.edu (W.W.C.); eperens@health.ucsd.edu (E.A.P.); eduolive812@gmail.com (E.A.O.); 2 Health Sciences Postgraduate Program, School of Medicine, Federal University of Minas Gerais (UFMG), Belo Horizonte 30130-100, MG, Brazil; 3 Institute of Biochemistry, Food Science and Nutrition, Hebrew University of Jerusalem, Rehovot 7610001, Israel; arieh.gertler@mail.huji.ac.il

**Keywords:** infantile nephropathic cystinosis, leptin, cachexia, adipose tissue browning, muscle wasting

## Abstract

Mice lacking the functional cystinosin gene (*Ctns^−/−^*), a model of infantile nephropathic cystinosis (INC), exhibit the cachexia phenotype with adipose tissue browning and muscle wasting. Elevated leptin signaling is an important cause of chronic kidney disease-associated cachexia. The pegylated leptin receptor antagonist (PLA) binds to but does not activate the leptin receptor. We tested the efficacy of this PLA in *Ctns^−/−^* mice. We treated 12-month-old *Ctns^−/−^* mice and control mice with PLA (7 mg/kg/day, IP) or saline as a vehicle for 28 days. PLA normalized food intake and weight gain, increased fat and lean mass, decreased metabolic rate and improved muscle function. It also attenuated perturbations of energy homeostasis in adipose tissue and muscle in *Ctns^−/−^* mice. PLA attenuated adipose tissue browning in *Ctns^−/−^* mice. PLA increased gastrocnemius weight and fiber size as well as attenuated muscle fat infiltration in *Ctns^−/−^* mice. This was accompanied by correcting the increased expression of muscle wasting signaling while promoting the decreased expression of myogenesis in gastrocnemius of *Ctns^−/−^* mice. PLA attenuated aberrant expressed muscle genes that have been associated with muscle atrophy, increased energy expenditure and lipolysis in *Ctns^−/−^* mice. Leptin antagonism may represent a viable therapeutic strategy for adipose tissue browning and muscle wasting in INC.

## 1. Introduction

Cystinosin is a lysosomal protein that functions as an active transporter for the export of cystine molecules out of the lysosome. Mutations in CTNS are responsible for cystinosis, an autosomal recessive lysosomal storage disease [1,2]. Infantile nephropathic cystinosis (INC) is a genetic form of chronic kidney disease (CKD) caused by mutations in the cystinosin (CTNS) gene [1], resulting in the accumulation of cystine crystals in cells and tissues [2]. Children with INC exhibit progressive Fanconi syndrome and CKD [3]. Common metabolic complications in patients with INC include loss of adipose tissue and muscle mass, which are associated with poor quality of life and mortality. There is no effective therapy for these complications [3,4].

Cachexia in CKD is a complex metabolic disorder driven by inflammation, which results in profound loss of adipose tissue and muscle mass [5]. Adipose tissue is a critical metabolic and secretory organ consisting of white adipose tissue (WAT) and brown adipose tissue (BAT). WAT functions as an energy-storing organ and BAT uses stored energy for heat production during thermogenesis [6]. Recent studies highlight the important role of WAT browning, a process characterized by a phenotypic switch from energy-storing white adipocytes to thermogenic brown-fat-like cells, in the development and progression of cachexia [7,8,9,10]. Leptin influences WAT browning. Leptin can directly influence adipocyte metabolism as adipocytes express the leptin receptor. Leptin can also indirectly influence adipocyte metabolism as adipocytes are sympathetically innervated and are insulin-sensitive [11]. Leptin is an important immunomodulatory cytokine and it is a pivotal regulator of energy homeostasis. Leptin receptors are found both centrally and peripherally [12,13]. Leptin crosses the blood–brain barrier and exerts its function by binding to its hypothalamic receptor and inhibits orexigenic signaling pathways as well as stimulates anorexigenic signaling pathways. Previously, we reported that modulation of the central melanocortin signaling by leptin is an important cause of CKD-associated cachexia [14]. The blocking of leptin activity may provide a novel therapeutic strategy for cachexia in CKD. The pegylated leptin receptor antagonist (PLA) binds but does not activate the leptin receptor [15,16]. PLA increases food intake and weight gain in mice [17]. We have shown that PLA ameliorates cachexia in CKD mice [18]. We hypothesize that leptin may be an important cause of INC-associated cachexia. In this study, we tested the effectiveness of PLA in *Ctns*^−/−^ mice, an established model of INC, in which we have previously demonstrated cachexia, characterized by adipose tissue browning and muscle wasting [19,20].

## 2. Materials and Methods

### 2.1. Study Design

This study was conducted in compliance with established guidelines and prevailing protocol (S01754) as approved by the Institutional Animal Care and Use Committee (IACUC) at the University of California, San Diego in accordance with the National Institutes of Health. PLA was prepared [17] and provided by the Hebrew University of Jerusalem. Twelve-month-old male mice were used for the study. A breeding pair of *Ctns^−/−^* mice was kindly provided by Professors Corinne Antignac and Stephanie Cherqui. Wild-type (WT) mice were purchased from the Jackson Laboratory. *Ctns^−/−^* mice and wild-type (WT) mice were on the same c57BL/6 genetic background. Mice were housed in 12:12 hour light-dark cycles with *ad libitum* access to mouse diet 5015 (LabDiet, catalog 0001328, with a metabolizable energy value of 3.59 kcal/gram) and water prior to the initiation of the experiment. We have performed the following two studies. Study 1—we evaluated the effects of PLA in *Ctns^−/−^* mice and WT mice. *Ctns^−/−^* mice and WT mice were given PLA (7 mg/kg/day, IP) or vehicle (normal saline), respectively. The study period was 28 days and all mice were fed *ad libitum*. We compared caloric intake and accompanying weight change in *Ctns^−/−^* mice and WT mice. Mice were housed in individual cages and had free access to the rodent diet. We measured the daily food intake and weight change for each mouse. The caloric intake for each mouse was calculated by multiplying total mouse diet consumption during the 28 days (in grams) with the metabolizable energy value of the diet (3.59 kcal/gram). The final result for energy intake is expressed as kcal/mouse/day. Study 2—we evaluated the effects of PLA in *Ctns^−/−^* mice, beyond nutritional stimulation by employing a pair-feeding strategy. *Ctns^−/−^* mice and WT mice were given PLA (7 mg/kg/day, IP) or vehicle (normal saline) for 28 days. Vehicle-treated *Ctns^−/−^* mice were fed *ad libitum* while all other groups of mice were fed the same amount of rodent diet based on the recorded food intake of vehicle-treated *Ctns^−/−^* mice. Each mouse was housed in an individual cage. We measured daily weight change for each mouse.

### 2.2. Body Composition Analysis

Body composition (for lean and fat content) was measured by quantitative magnetic resonance analysis (EchoMRI-100^TM^, Echo Medical System) [19]. All measurements were made during the light phase (0900-1900). Actual procedures were performed according to the manufacturer’s instruction. Contents of whole-body fat, lean mass, free water, and total body water were calculated.

### 2.3. Resting Metabolic Rate

Indirect calorimetry was performed in mice using Oxymax calorimetry (Columbus Instruments). Oxygen (VO_2_) and carbon dioxide (VCO_2_) consumption were measured. The respiratory exchange ratio (RER) was calculated as the quotient VCO_2_/VO_2_. Energy expenditure was measured as a production of kilocalorie of heat and was calculated as Caloric Value (CV) × VO_2_ where CV is 3.815 + 1.232 × RER. Final resting metabolic rate (RER) in an individual mouse is expressed as kcal/mouse/day [21].

### 2.4. Mouse Muscle Function

Measurements of muscle function were recorded at the end of the study. All measurements were conducted by one trained investigator. For forelimb strength, holding the mice by the tail, the front feet were allowed to grip a grate, and then they were pulled from the grate, generating a force measured by the force transducer (Model 47106, UGO Basile). Five measurements were taken, three days consecutively, with the first day used as acclimatization and not included in final data analysis. The average of the measurements was used in the data analysis [19,20]. Grip strength was reported in gram of strength per gram of body mass. Rotarod performance was used to assess neuromuscular coordination at the conclusion of the study. All mice underwent three days of acclimatization prior to data collection. Mice were placed on the rod in the identical forward direction, and then the rotarod performance tool (model RRF/SP, Accuscan Instrument) was started at 0 rpm and increased to 40 rpm at 0.4 rpm/s, after a 60 s acclimatization period at 4 rpm. The latency to fall from the rod was recorded in seconds with a maximum time of 300 s. This procedure was repeated for a total of six trials (3 trials per day with > 2 h of rest time between trials) over three days, and the average of the trials was used in the data analysis. Rotarod performance was reported as latency to fall in seconds [19,20].

### 2.5. Serum and Blood Chemistry

BUN and bicarbonate concentration of serum were measured (Supplemental Table S1). Serum creatinine was analyzed by the LC-MS/MS method [22]. Serum leptin concentration was also measured.

### 2.6. Protein Assay for Muscle and Adipose Tissue

Portion of the right gastrocnemius muscle of mice, inguinal WAT and intercapsular BAT, were processed in a homogenizer tube (USA Scientific, catalog 1420-9600) containing ceramic beads (Omni International, catalog 19-646) using a Bead Mill Homogenizer (Omni International). Protein concentration of tissue homogenate was assayed using a Pierce BAC Protein Assay Kit (Thermo Scientific, catalog 23227). Uncoupling (UCP) protein content as well as adenosine triphosphate (ATP) concentration in adipose tissue and muscle homogenates were assayed (Supplemental Table S1).

### 2.7. Gastrocnemoius Weight, Fiber Size and Fatty Infiltration

The left gastrocnemius muscle of mice was dissected at the end of the study. Wet weight of left gastrocnemius muscle was recorded. Subsequently, we processed the dissected gastrocnemius according to an established protocol and measured muscle fiber cross-sectional area, using ImageJ software (https://rsbweb.nih.gob/ij/, accessed on 23 March 2021) [19,20]. We also quantified fatty infiltration in skeletal muscle. Dissected muscle samples were incubated with Oil Red O (Oil Red O Solution, catalog number O1391-250 mL, Sigma Aldrich, St. Louis, MO, USA) [23]. Detailed procedures for Oil Red O staining were in accordance with published protocol. Acquisition and quantification of images were analyzed using ImageJ software (https://rsbweb.nih.gob/ij/, accessed on 23 March 2021 [24].

### 2.8. Muscle RNAseq Analysis

Previously, we performed RNAseq analysis on gastrocnemius muscle mRNA in 12-month-old *Ctns^−/−^* mice versus age-appropriate WT mice [20]. Detailed procedures for mRNA extraction, purification and subsequent construction of cDNA libraries as well as analysis of gene expression were published [20]. We then performed Ingenuity Pathway Analysis enrichment tests for those differentially expressed muscle genes in *Ctns^−/−^* mice versus WT mice, focusing on pathways related to energy metabolism, skeletal and muscle system development and function, and organismal injury and abnormalities. We identified the top 20 differentially expressed muscle genes in *Ctns^−/−^* versus WT mice. In this study, we performed qPCR analysis for those top 20 differentially expressed gastrocnemius muscle genes in the different experimental groups.

### 2.9. Quantative Real-Time PCR

A portion of the right gastrocnemius muscle of mice, inguinal WAT and intercapsular BAT, was processed in a homogenizer tube (USA Scientific, catalog 1420-9600) containing ceramic beads (Omni International, catalog 19-646) using a Bead Mill Homogenizer (Omni International). Total RNA from gastrocnemius and adipose tissues was isolated using TriZol (Life Technology). Total RNA (3 µg) was reverse transcribed to cDNA with SuperScript III Reverse Transcriptase (Invitrogen). Quantitative real-time RT-PCR of target genes was performed using KAPA SYBR FAST qPCR kit (KAPA Biosystems) [19,20]. Glyceraldehyde−3-phosphate dehydrogenase (GAPDH) was used as an internal control. Expression levels were calculated according to the relative 2^−ΔΔCt^ method. All primers are listed (Supplemental Table S2).

### 2.10. Statistics

Statistical analyses were performed using GraphPad Prism version 9.1.1. All data are presented as mean ± S.E.M. For comparison of the means between two groups, data were analyzed by Student’s 2-tailed *t*-test. Differences of the means for more than two groups containing two variables were analyzed using 2-way ANOVA. *Post-hoc* analysis was performed with Tukey’s test. A *p*-value less than 0.05 was considered significant.

## 3. Results

### 3.1. Pegylated Leptin Antagonist (PLA) Normalizes Food Intake and Improves Weight Gain in Ctns^−/−^ Mice

**Figure 1:**
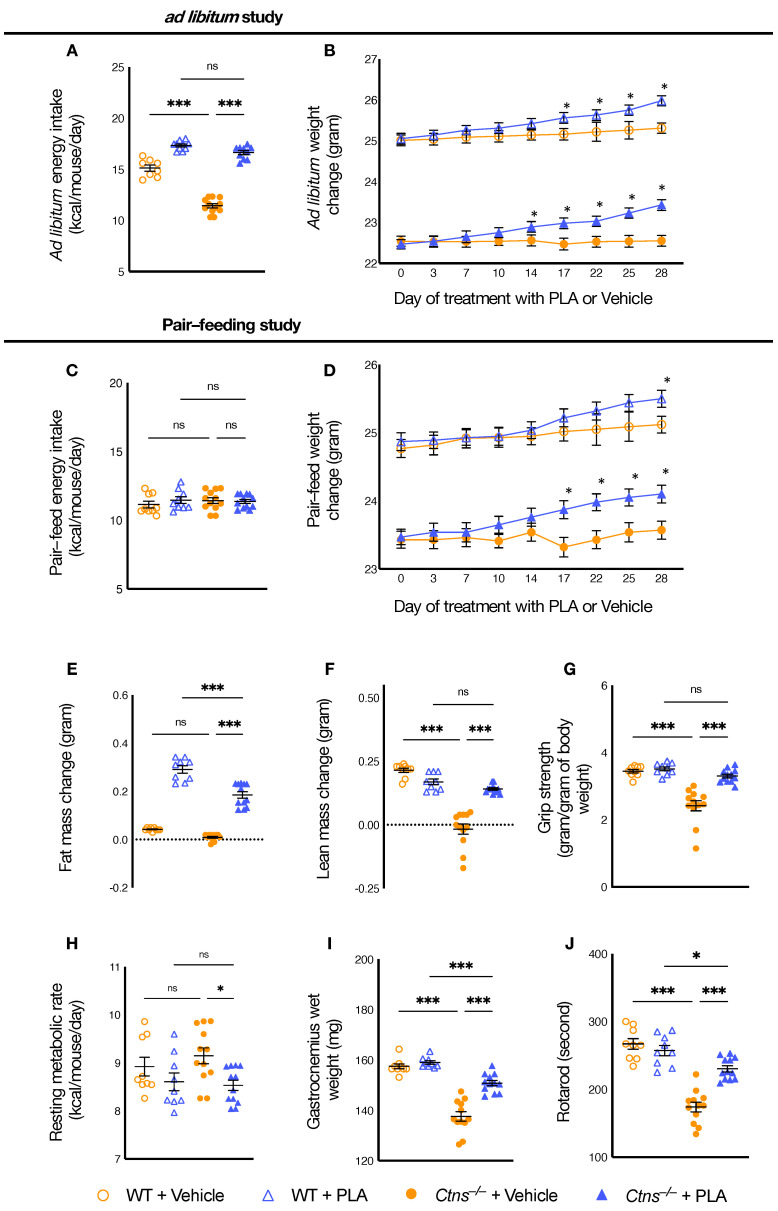
PLA attenuates cachexia in *Ctns^−/−^* mice. We have performed the following two studies. *Ctns^−/−^* and WT mice were given PLA (7 mg/kg/day, IP) or vehicle (normal saline), respectively. The study period was 28 days and all mice were fed *ad libitum*. We calculated *ad libitum* caloric intake (**A**) and recorded weight change in mice (**B**). In another experiment, to assess the beneficial effects of PLA beyond its nutritional effects, we employed a pair-feeding strategy. *Ctns^−/−^* and WT mice were given PLA (7 mg/kg/day, IP) or vehicle (normal saline) for 28 days. Vehicle-treated *Ctns^−/−^* mice were given an *ad libitum* amount of food whereas other groups of mice were given an equivalent amount of food (**C**). Weight gain, fat and lean content, resting metabolic rate (RME), left gastrocnemius wet weight and in vivo muscle function (grip strength and rotarod) were measured in mice (**D**–**J**). Data are expressed as mean ± SEM. For weight change in mice (**B**,**D**), results of vehicle-treated WT mice were compared to PLA-treated WT mice while vehicle-treated *Ctns^−/−^* mice were compared to PLA-treated *Ctns^−/−^* mice. For other data (**A**,**C**,**E**–**J**), results of vehicle-treated *Ctns^−/−^* mice were compared to vehicle-treated WT mice while results of PLA-treated *Ctns^−/−^* mice were compared to those of PLA-treated WT mice. In addition, results of PLA-treated *Ctns^−/−^* mice were compared to vehicle-treated *Ctns^−/−^* mice and specific *p*-values are shown above the bar. *ns* signifies not significant, * *p* < 0.05, *** *p* < 0.001.

We studied the dietary effects of PLA in *Ctns^−/−^* mice. PLA (7 mg/kg/day, IP) or vehicle (normal saline) was administrated to 12-month-old *Ctns^−/−^* and WT mice for 28 days. Mice were fed *ad libitum*. PLA ameliorated anorexia in *Ctns^−/−^* mice. Average daily calorie intake was normalized in PLA-treated *Ctns^−/−^* mice relative to PLA-treated WT mice (Figure 1A). Significant weight gain in PLA-treated *Ctns^−/−^* mice relative to vehicle-treated *Ctns^−/−^* mice was observed at day 14, and the trend remained significant for the rest of the study (Figure 1B).

### 3.2. PLA Attenuates Cachexia in Ctns^−/−^ Mice

To investigate the pharmacological consequences of inhibiting the leptin receptor in *Ctns^−/−^* mice beyond appetite stimulation and their consequent body weight gain, we employed a food restrictive strategy. *Ctns^−/−^* and WT mice were given PLA (7 mg/kg/day, IP) or vehicle for 28 days. Vehicle-treated *Ctns^−/−^* mice were fed *ad libitum*. Daily *ad libitum* caloric intake for vehicle-treated *Ctns^−/−^* mice was measured. Subsequently, PLA-treated *Ctns^−/−^* mice, PLA-treated WT, and vehicle-treated WT mice were given the equivalent amount of energy intake as vehicle-treated *Ctns^−/−^* mice (Figure 1C). At the end of the study, mice were sacrificed, and we measured serum and blood chemistry of mice (Table 1). Serum concentration of leptin was significantly increased in *Ctns^−/−^* mice relative to control mice. PLA did not influence serum leptin levels in *Ctns^−/−^* mice. We showed that PLA attenuated or normalized weight gain, fat and lean mass content, resting metabolic rate, gastrocnemius weight, and in vivo muscle function (grip strength and rotarod activity) in *Ctns^−/−^* mice relative to PLA-treated WT mice (Figure 1D–J).

### 3.3. PLA Attenuates Aberrant Adipose Tissue and Skeletal Muscle Energy Homeostasis in Ctns^−/−^ Mice

**Figure 2:**
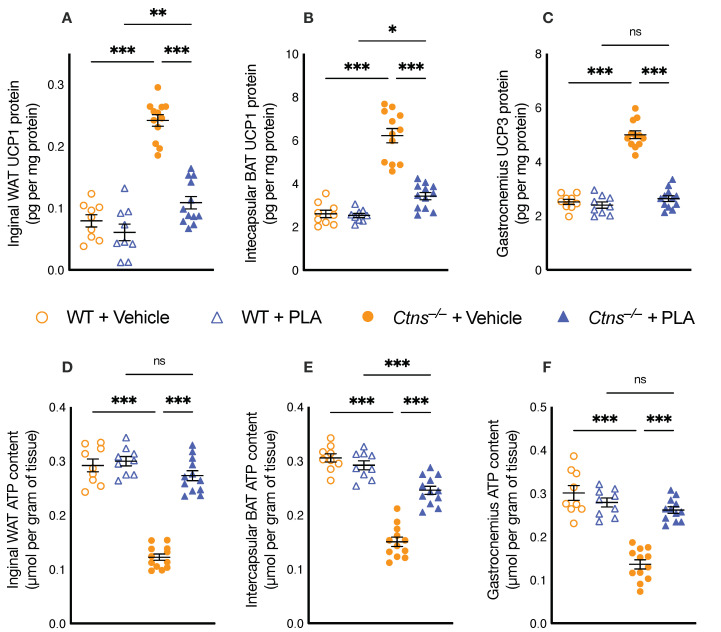
PLA ameliorates energy homeostasis in adipose tissue and skeletal muscle in *Ctns^−/−^* mice. UCP and ATP content of inguinal WAT (**A**,**D**), intercapsular BAT (**B**,**E**) and gastrocnemius muscle (**C**,**F**) were measured. Results of vehicle-treated Ctns−/− mice were compared to vehicle-treated WT mice while results of PLA-treated *Ctns^−/−^* mice were compared to those of PLA-treated WT mice. In addition, results of PLA-treated *Ctns^−/−^* mice were compared to vehicle-treated *Ctns^−/−^* mice and specific *p*-values are shown above the bar. *ns* signifies not significant, * *p* < 0.05, ** *p* < 0.01, *** *p* < 0.001.

Metabolism of adipose tissue and skeletal muscle influence basal metabolic rate and energy expenditure, which can substantially affect whole body metabolism and weight gain. We studied the effects of PLA on adipose tissue and skeletal muscle energy homeostasis in *Ctns^−/−^* mice. Protein content of UCPs in WAT, BAT as well as gastrocnemius was significantly higher in *Ctns^−/−^* mice (Figure 2). Inversely, ATP content in WAT, BAT and gastrocnemius was significantly lower in *Ctns^−/−^* mice. We showed that PLA attenuated UCPs and ATP content in adipose tissue and muscle in *Ctns^−/−^* mice.

### 3.4. PLA Attenuates White Adipose Tissue Browning in Ctns^−/−^ Mice

**Figure 3:**
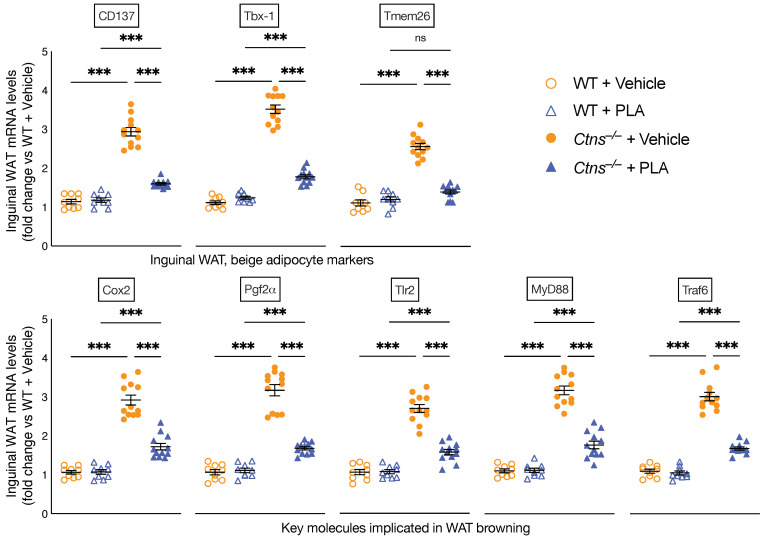
PLA attenuates adipose tissue browning in *Ctns^−/−^* mice. Gene expression of beige adipocyte markers (CD137, Tb-1 and Tmem26) in inguinal WAT was measured by qPCR. Gene expression of the Cox2 signaling pathway and toll like receptor pathway (Cox2, Pgf2α, Tlr2, Myd88 and Traf6) in inguinal WAT was also measured. Final results were expressed in arbitrary units, with one unit being the mean level in vehicle-treated WT mice. In addition, results of PLA-treated *Ctns^−/−^* mice were compared to vehicle-treated *Ctns^−/−^* mice and specific *p*-values are shown above the bar. *ns* signifies not significant, *** *p* < 0.001.

Recent data suggest that browning of white adipose tissue contributes to energy wasting in cachexia. White, beige and brown adipocytes are distinct but often occur mixed together within individual depots. We studied the effects of PLA on white adipose tissue browning by measuring the expression of beige adipocyte cell surface markers (CD137, Tmem26, and Tbx1) in inguinal WAT in *Ctns^−/−^* mice. We showed that PLA attenuated inguinal WAT expression of CD137, Tmem26, and Tbx1 in *Ctns^−/−^* mice (Figure 3). UCP1 is exclusively expressed in brown adipocytes. We showed that PLA attenuated UCP1 protein content, which was significantly increased in inguinal WAT of *Ctns^−/−^* mice (Figure 2). We further investigated the effects of PLA on expression of key molecules implicated in adipocyte tissue browning in *Ctns^−/−^* mice. Cox2 is the rate-limiting enzyme for synthesis of prostaglandins and increased expression of Cox2/Pgf2α induces *de novo* browning recruitment in WAT and facilitates systemic energy expenditure. Activation of toll-like receptor Tlr2 and their adaptor molecules such as MyD88 and Traf6 have been implicated in white adipocyte browning. We showed that PLA attenuated inguinal WAT expression of Cox2, Pgf2α as well as Tlr2, MyD88 and Traf6 in *Ctns^−/−^* mice (Figure 3).

### 3.5. PLA Attenuates Muscle Wasting Signaling Pathways in Ctns^−/−^ Mice

**Figure 4:**
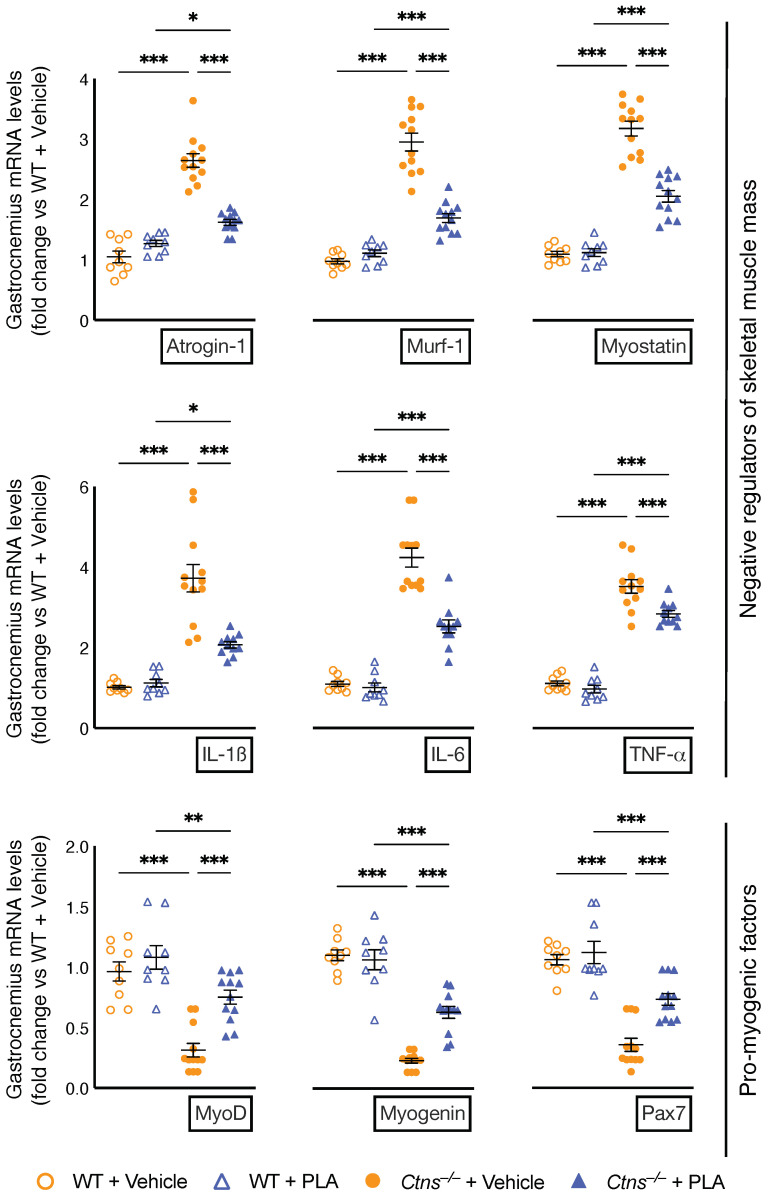
PLA attenuates signaling pathways implicated in muscle wasting in *Ctns^−/−^* mice. Gastrocnemius muscle expression of negative regulators of skeletal muscle mass (Atrogin-1, Murf-1, Myostatin, IL-1β, IL-6 and TNF-α) as well as pro-myogenic factors (MyoD, Myogenin and Pax7) were measured by qPCR. Final results were expressed in arbitrary units, with one unit being the mean level in vehicle-treated WT mice. In addition, results of PLA-treated *Ctns^−/−^* mice were compared to vehicle-treated *Ctns^−/−^* mice and specific *p*-values are shown above the bar. * *p* < 0.05, ** *p* < 0.01, *** *p* < 0.001.

PLA ameliorated muscle regeneration and myogenesis by attenuating expression of negative regulators of skeletal muscle mass (Atrogin-1, Murf-1 and Myostatin) and pro-myogenic factors (MyoD, Myogenin and Pax-7) in *Ctns^−/−^* mice (Figure 4). Inflammatory cytokines induce muscle atrophy. PLA attenuated gastrocnemius expression of inflammatory cytokines (IL-1β, IL-6 and TNFα) in *Ctns^−/−^* mice.

### 3.6. PLA Increases Muscle Fiber Size and Attenuates Muscle Fat Infiltration in Ctns^−/−^ Mice

**Figure 5:**
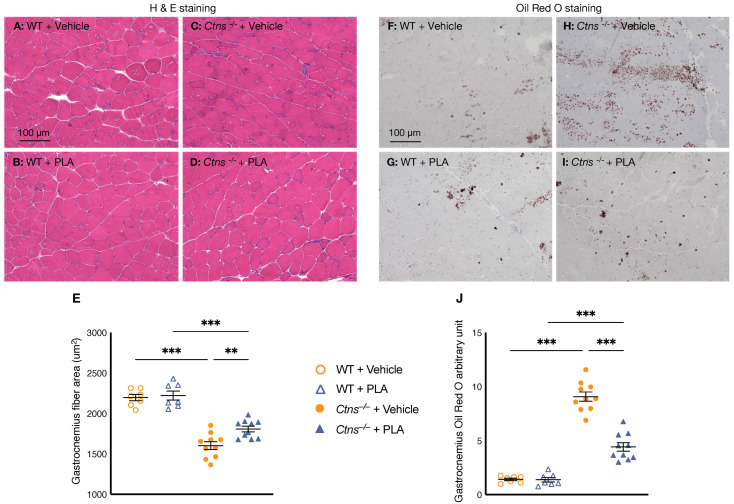
PLA increases muscle fiber size and attenuates muscle fat infiltration in *Ctns^−/−^* mice. Representative photomicrographs of the gastrocnemius with H&E staining (**A**–**D**). Average gastrocnemius cross-sectional area was measured (**E**). Visualization of the quantification of fatty infiltration by Oil Red O analysis in the gastrocnemius muscle (**F**–**J**). Final results were expressed in arbitrary units, with one unit being the mean staining intensity in vehicle-treated WT mice. In addition, results of PLA-treated *Ctns^−/−^* mice were compared to vehicle-treated *Ctns^−/−^* mice and specific *p*-values are shown above the bar. ** *p* < 0.01, *** *p* < 0.001.

We also studied the effect of PLA on skeletal muscle morphology in *Ctns^−/−^* mice. We showed that PLA increased average cross-sectional area of the gastrocnemius in *Ctns^−/−^* mice (Figure 5A–E). Fatty infiltration of the skeletal muscle is a common and important feature of many myopathies. We showed that PLA attenuated fatty infiltration in skeletal muscle in *Ctns^−/−^* mice (Figure 5F–J).

### 3.7. Muscle Transcriptome Study by RNAseq Analysis

**Figure 6:**
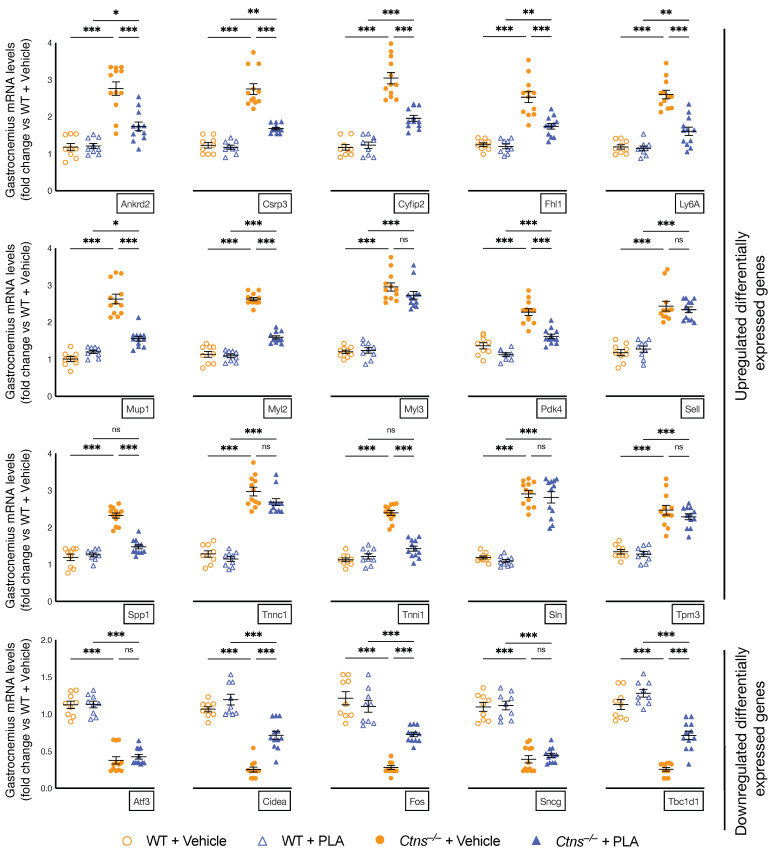
PLA attenuates expression of gastrocnemius muscle genes in *Ctns^−/−^* mice. PLA attenuated or normalized (Ankrd2, Csrp3, Cyfip2, Fhl1, Ly6A, Mup1, Myl1, Pdk4, Spp1 and Tnni1) as well as (Cidea, Fos and Tbc1d1) muscle gene expression in *Ctns^−/−^* mice relative to PLA-treated WT mice. Nonsignificant changes were observed in Myl3, Sell, Sln, Tnnc1 and Tpm3 as well as Atf3 and Sncg. Gastrocnemius muscle expression of targeted molecules was measured by qPCR. Final results were expressed in arbitrary units, with one unit being the mean level in vehicle-treated WT mice. In addition, results of PLA-treated *Ctns^−/−^* mice were compared to vehicle-treated *Ctns^−/−^* mice and specific *p*-values are shown above the bar. *ns* signifies not significant, * *p* < 0.05, ** *p* < 0.01, *** *p* < 0.001.

Previously, we performed gastrocnemius RNAseq analysis between 12-month-old *Ctns^−/−^* and control mice and identified top differentially expressed genes which have been associated with energy metabolism, skeletal and muscular system development and function, nervous system development and function as well as organismal injury and abnormalities [20]. In this study, we examined the effects of PLA on gastrocnemius expression of the top 20 differentially expressed genes previously identified [20]. Nonsignificant changes were observed in Myl3, Sell, Sln, Tnnc1 and Tpm3 as well as Atf3 and Sncg. Importantly, PLA attenuated or normalized (Ankrd2, Csrp3, Cyfip2, Fhl1, Ly6A, Mup1, Myl1, Pdk4, Spp1 and Tnni1) as well as (Cidea, Fos and Tbc1d1) muscle gene expression in *Ctns^−/−^* mice (Figure 6). Table 2 summarizes the functional significance of these 13 differentially expressed muscle genes.

## 4. Discussion

In this study, we tested the effectiveness of a leptin receptor antagonist in *Ctns^−/−^* mice, an established model of INC. Our results suggest that this PLA may work peripherally as well as centrally. Previous studies confirm that this PLA crosses the blood–brain barrier. Peripheral injection of this PLA inhibits the binding of CNS leptin to its receptor [15]. Serum concentration of leptin was significantly increased in 12-month-old *Ctns^−/−^* mice versus age-appropriated control mice. We demonstrated that PLA treatment did not influence serum concentration of leptin in mice. Serum concentration of leptin was not different in PLA-treated *Ctns^−/−^* mice versus vehicle-treated *Ctns^−/−^* mice (Table 1).

PLA improved caloric intake and weight gain in *Ctns^−/−^* mice (Figure 1A,B). Our findings also emphasize the favorable effects of PLA beyond appetite stimulation and accompanied weight gain. In food-restricted experiments, vehicle-treated *Ctns^−/−^* mice were fed *ad libitum* while PLA-treated *Ctns^−/−^* mice, PLA-treated WT mice and vehicle-treated WT mice were fed an equivalent amount of calories as vehicle-treated *Ctns^−/−^* mice (Figure 1C). The cachexia phenotype was attenuated or normalized in PLA-treated *Ctns^−/−^* mice relative to WT mice (Figure 1D–J). The results from this study are consistent with several prior studies. Leptin antagonism attenuated leptin-induced weight loss, loss of fat content, and anorexia [41,42]. Previously, we have shown that PLA treatment attenuated cachexia in CKD mice [18].

The basal metabolic rate is responsible for up to 80% of the daily caloric expenditure in a human individual [43]. However, for brown/beige adipose physiology, the mouse data may not be predictive of human data [44]. Supplementation of leptin increases energy expenditure through activation of BAT thermogenesis in mice where leptin is being added back to the leptin-deficient state [45]. In this study, PLA attenuated the elevated resting metabolic rate in *Ctns^−/−^* mice (Figure 1H). Adipose tissue UCP1 may influence but not be essential for adaptive adrenergic non-shivering thermogenesis [46]. UCPs modulate energy homeostasis by ‘uncoupling’ ATP generation, thereby dissipating the mitochondrial proton gradient for ATP synthesis and generating heat [47,48]. We demonstrated that PLA treatment attenuated aberrant adipose tissue and muscle UCP and ATP content in *Ctns^−/−^* mice (Figure 2). Scarpace PJ et al. revealed that leptin increased BAT UCP mRNA content in both *ad libitum*-fed and food-restricted rodents [49]. Interestingly, Okamatsu-Ogura et al. demonstrated that UCP1 enhances leptin activity in the hypothalamus [50].

Leptin suppresses appetite and increases energy expenditure via activation of pro-opiomelanocortin neurons, which is dependent upon some degree of ‘cross-talk’ between leptin signaling and other inflammatory cytokines. Type I cytokine receptors include leptin receptor and IL-6 receptor [51]. Circulating concentrations of leptin decrease in the fasting state while they increase during the postprandial phase that follows. These changes are associated with expression of hypothalamic IL-1β mRNA levels, indicating that leptin can influence inflammation centrally [52]. The inflammatory cytokines TNFα and IL-1 increased the concentration of circulating leptin and adipose tissue expression of leptin in hamsters [53]. These changes were accompanied by a decline in food intake [54]. The notion of cross-talking between leptin signaling and inflammatory cytokines is further supported through experiments whereby peripheral administration of leptin increased hypothalamic expression of IL-1 and suppressed food intake while central administration of the IL-1 receptor antagonist abolished the leptin-induced anorexia [55].

Leptin is important for the pathogenesis of disease-associated cachexia such as cancer cachexia, chronic heart failure-induced cachexia, pulmonary cachexia, aging-associated cachexia and CKD-associated cachexia [56]. For patients with CKD, it is possible to remove circulating leptin using super-flux polysulfone dialyzers. van Tellingen et al. utilized said approach, which significantly reduced the circulating concentration of leptin; however, they did not assess other important metabolic parameters including appetite or body composition. Due to this, the usefulness of such therapy is yet to be proven [57,58]. Inflammatory cytokines such as leptin, IL-1β, IL-6 and TNFα are increased in CKD-associated cachexia [5]. Leptin and these inflammatory cytokines modulate hypothalamic feedback mechanisms and contribute to CKD-associated cachexia. The crosstalk between leptin signaling and other inflammatory cytokines could explain why patients do not exhibit increased appetite or lower energy expenditure, despite decreased levels of leptin in many disease-associated cachexic states such cancer, COPD, and aging. In this aspect, leptin receptor antagonists, such as this PLA, could provide a novel and superior approach than clearance of circulating leptin for cachexia in INC.

We demonstrated that PLA treatment attenuated browning of adipose tissue in *Ctns^−/−^* mice. Browning of adipose tissue, also referred to as “beiging” or “briting” is critical to the hypermetabolic state. Browning of adipose tissue has been implicated in cachexia [7,8,9,10,59]. Recent studies have demonstrated that adipose tissue browning precipitates muscle wasting observed in cachectic patients [60,61]. Reducing the severity of the browning ameliorates cachexia [62]. Browning was defined by the detection of WAT UCP1 protein (Figure 2) and increased expression of beige adipose cell markers CD137, Tmem and Tbx1 in *Ctns^−/−^* mice (Figure 3). Many biomarkers have been associated with the presence of beige adipocytes and WAT browning. We recognize that there are new in vivo studies that call into the question the role of CD137, Tmem26 and Tb-1 as reliable beige markers [63]. However, these same studies acknowledge the possibility of a loss of function for these markers *in vivo*. Furthermore, those studies rely on cold exposure to induce browning which does not properly mimic the systemic dysfunction of the cachexia phenotype [64,65]. Importantly, we showed that there is a distinct elevated expression of WAT CD137, Tmem26 and Tb-1 in *Ctns^−/−^* mice. Moreover, we showed that mRNA expression of these markers was attenuated by PLA treatment in *Ctns^−/−^* mice (Figure 3).

Multiple pathways have been implicated for biogenesis of browning. Cox2/Pgf2α induces *de novo* browning recruitment in WAT. Activation of Tlr2, MyD88 and Traf6 have been implicated in browning [61]. We demonstrated that PLA treatment normalized inguinal WAT Cox2, Pgf2α, along with important inflammatory molecules (Tlr2, MyD88 and Trap6) expression in *Ctns^−/−^* mice (Figure 3). Recent data suggest that leptin promotes WAT browning by inhibiting the Hh signaling pathway [66].

Leptin induces muscle wasting in a zebrafish model of cancer cachexia [67]. Elevated serum concentration of leptin was associated with low density skeletal muscle area at the mid-thigh in postmenopausal women [68]**.** Fatty infiltration of the skeletal muscle is common and has been associated with muscle weakness and impaired physical function across a diverse set of many myopathies [69,70,71,72,73]. PLA treatment improved lean mass content, gastrocnemius wet weight and muscle function in *Ctns^−/−^* mice (Figure 1F,G,I,J). In addition, PLA increased muscle fiber size and reduced muscle fat infiltration in *Ctns^−/−^* mice (Figure 5).

We studied the expression of molecules regulating skeletal muscle mass in *Ctns^−/−^* mice. PLA decreases mRNA expression of negative regulators of skeletal muscle mass (Atrogin-1, Murf-1, Myostatin and inflammatory cytokines IL-1β, IL-6 and TNFα) and increased expression of pro-myogenic factors (MyoD, Myogenin and Pax-7) in *Ctns^−/−^* mice (Figure 4). The transcription factors Pax-3 and Pax-7 are primary contributors to skeletal muscle repair and growth. Pax-3 and Pax-7 function upstream of MyoD and myogenin [74]. MyoD, in conjunction with myogenic factor 5 (Myf5), is necessary for determination of myogenic precursors. Myogenin is a downstream target of MyoD and is responsible for regulating myoblast development into myocytes and myotubes [75].

Finally, we evaluated the muscle transcriptome by RNAseq analysis. PLA treatment in *Ctns^−/−^* mice attenuated 10 of the 15 upregulated genes and three of the five downregulated genes (Table 2). Increased muscle expression of Ankrd, Csrp3, Myl2, Spp1 and Tnni1 as well as downregulated expression of Fos correspond to a decrease ability for muscle regeneration and muscle function [24,25,26,31,32,34,35]. Upregulated expression of Mup1 and downregulated expression of Cidea is related to an increase in muscle energy metabolism and lipolysis [31,37]. Increased expression of Ly6a and Cyfip2 promote fibrosis and apoptosis, respectively [28,30]. Decreased expression of Tbc1d1 impairs glucose transport in skeletal muscle [39] and is associated with follistatin-induced muscle wasting [40]. Increase Pdk4 expression is a biomarker for muscle energy deprivation [33]. Furthermore, increased expression of muscle Fhl1 is associated with muscle atrophy and muscle weakness [29].

## 5. Conclusions

**Figure 7:**
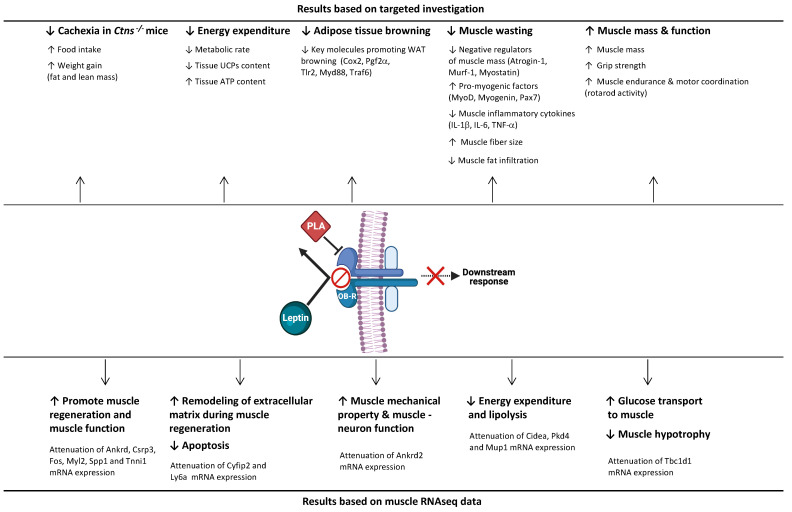
Summary of the beneficial effects of a leptin receptor antagonist on cachexia, energy expenditure, adipose tissue browning and muscle wasting in *Ctns^−/−^* mice. Created with BioRender.com, accessed 18 May 2021.

We employed a pharmacological approach and demonstrated significant beneficial effects of leptin receptor blockade on cachexia in *Ctns^−/−^* mice via multiple cellular mechanisms (Figure 7). Leptin receptor antagonism represents a novel therapeutic strategy for cachexia in patients with INC by attenuating adipose tissue browning and muscle wasting.

## Figures and Tables

**Table 1:** Serum and blood chemistry of mice. Twelve-month-old *Ctns^−/−^* and WT mice were treated with PLA (7 mg/kg/day, IP) or vehicle (normal saline) for 28 days. Four groups of mice were included: WT + Vehicle, WT + PLA, *Ctns^−/−^* + Vehicle and *Ctns^−/−^* + PLA. *Ctns^−/−^* + Vehicle mice were fed *ad libitum* whereas WT + Vehicle, WT + PLA and *Ctns^−/−^* + PLA mice were pair-fed to that of *Ctns^−/−^* + Vehicle mice. Data are expressed as mean ± SEM. Results of *Ctns^−/−^* + Vehicle mice were compared to those of WT + Vehicle mice whereas results of *Ctns^−/−^* + PLA mice were compared to those of WT + PLA mice. * *p* < 0.05.

	WT + Vehicle (*n* = 9)	WT + PLA (*n* = 9)	*Ctns^−/−^* + Vehicle (*n* = 12)	*Ctns^−/−^* + PLA (*n* = 12)
BUN (mg/dL)	29.5 ± 4.6	32.4 ± 3.1	59.8 ± 6.7 *	74.5 ± 9.4 *
Creatinine (mg/dL)	0.09 ± 0.02	0.12 ± 0.04	0.19 ± 0.02 *	0.21 ± 0.04 *
Bicarbonate (mmol/L)	28.2 ± 2.2	27.6 ± 2.1	27.3 ± 1.8	27.3 ± 1.1
Leptin (ng/mL)	2.6 ± 0.3	2.8 ± 0.5	4.6 ± 0.4 *	5.2 ± 0.3 *

**Table 2:** PLA normalizes or attenuated expression of important muscle genes in *Ctns^−/−^* mice. Functional significance for each of these differentially expressed muscle genes is listed. DEG, differential expressed genes.

Upregulated DEG	Functional Significance & Reference
Ankrd2	implicated in mechanical stretch of skeletal muscle [25,26]
Csrp3	associated with skeletal muscle dystrophy [27]
Cyfip2	associated with muscle atrophy [28]
Fhl1	activates myostatin signaling and promotes atrophy in skeletal muscle [29]
Ly6a	associated with remodeling of the extracellular matrix during skeletal muscle regeneration [30]
Mup1	increases energy expenditure in skeletal muscle [31]
Myl2	associated with muscle cycling kinetics [32,33]
Pdk4	associated with skeletal muscle energy deprivation via a FOXO1-dependent pathway [34]
Spp1	shares molecular network with myostatin and inhibits muscle regeneration [35]
Tnni1	regulates straited muscle contraction [36] implicated in cardiomyopathy pathogenesis and age-related skeletal muscle wasting [36]
Downregulated DEG	
Cidea	increases metabolic rates, lipolysis in brown adipose tissue and higher core temperature [37]
Fos	associated with decreased skeletal muscle regeneration [38]
Tbc1d1	impairs glucose transport in skeletal muscle [39] associated with follistatin-induced muscle wasting [40]

## Data Availability

The authors confirm that the data supporting the findings of this study are available within the article and its Supplementary Materials. Additional raw data supporting the findings of this study are available from the corresponding author (R.H.M.) on request.

## Supplementary Materials

The following are available online at https://www.mdpi.com/article/10.3390/cells10081954/s1, Table S1: Immunoassay information for blood and serum chemistry, muscle adenosine triphosphate content as well as muscle and adipose tissue protein analysis; Table S2: PCR primer information.
