# Supplementary material for: A Leptin Receptor Antagonist Attenuates Adipose Tissue Browning and Muscle Wasting in Infantile Nephropathic Cystinosis-Associated Cachexia
Source: Cells. 2021 Jul 31;10(8):1954. doi: 10.3390/cells10081954 (PMC8393983; doi:10.3390/cells10081954)
Supplement: Supplementary file 1 [file cells-10-01954-s001.zip › cells-1248866-supplementary.pdf]

Supplemental data

Article

Leptin receptor antagonist attenuates adipose tissue browning and muscle wasting in infantile nephropathic cystinosis-associated cachexia

Alex Gonzalez[1,#], Wai W Cheung [1,#], Elliot Perens[1], Eduardo A Oliveira[1,2], Arieh Gertler[3], Robert H Mak[1,*]

1 Pediatric Nephrology, Rady Children's Hospital San Diego, University of California, San Diego, La Jolla, USA
2 Health Sciences Postgraduate Program, School of Medicine, Federal University of Minas Gerais (UFMG), Belo Horizonte, MG 30130-100, Brazil
3 Institute of Biochemistry, Food Science and Nutrition, Hebrew University of Jerusalem, Rehovot, Israel
* Correspondence: romak@health.ucsd.edu; Tel.: +1 858 822 6717; Fax: +1 858 822 6776
\# These authors contributed equally to this work.

Supplemental Table S1: Immunoassay information for blood and serum chemistry, muscle adenosine triphosphate content as well as muscle and adipose tissue protein analysis.

Blood & Serum chemistry	Assay information
Bicarbonate & BUN	VetScan Comprehensive Diagnostic Profile, Abaxix, 500-0038
Creatinine	LC-MS/MS method (reference)
Leptin	Invitrogen, Mouse Leptin ELISA kit, catalog number KMC 2281

Muscle & adipose tissue	Assay information
ATP Assay Kit (Colorimetric / Fluorometric)	Abcam, ab83355
Mouse UCP1 ELISA kits	Aviva Systems Biology, OKCD02970
Mouse UCP3 ELISA kits	Aviva Systems Biology, OKEH05259

Supplemental Table S2: PCR primer information.

Gene	Forward primer sequence	Reverse primer sequence
Ankrd2	TGGACATGCTAGTGCTAGAGG	CGCTTTTTCTGCTTGCGTTTT
Atf3	GAGGATTTTGCTAACCTGACACC	TTGACGGTAACTGACTCCAGC
Atrogin-1	CAGCTTCGTGAGCGACCTC	GGCAGTCGAGAAGTCCAGTC
Cidea	TGACATTCATGGGATTGCAGAC	GGCCAGTTGTGATGACTAAGAC
CD137	CGTGCAGAACTCCTGTGATAAC	GTCCACCTATGCTGGAGAAGG
Cox2	AACCCAGGGGATCGAGTGT	CGCAGCTCAGTGTTTGGGAT
Csrp3	GGGGGAGGTGCAAAATGTG	CAGGCCATGCAGTGGAAACA
Cyfip2	ATGACCACCCACGTCACTTTG	CCTGTCCTCGAAGTTCGTGTC
Fhl1	GACTGCCGCAAGCCCATAA	CCAAGGGGTGAAGGCACTT
Fos	TTGAGCGATCATCCCGGTC	GCGTGAGTCCATACTGGCAAG
IL-1β	GCAACTGTTCCTGAACTCAACT	ATCTTTTGGGGTCCGTCAACT
IL-6	TAGTCCTTCCTACCCCAATTTCC	TTGGTCCTTAGCCACTCCTTC
Ly6a	AGGAGGCAGCAGTTATTGTGG	CGTTGACCTTAGTACCCAGGA
Mup1	GAAGCTAGTTCTACGGGAAGGA	AGGCCAGGATAATAGTATGCCA
Murf-1	GTGTGAGGTGCCTACTTGCTC	GCTCAGTCTTCTGTCCTTGGA
MyD88	TCATGTTCTCCATACCCTTGGT	AAACTGCGAGTGGGGTCAG
Myl2	ATCGACAAGAATGACCTAAGGGA	ATTTTTCACGTTCACTCGTCCT
Myl3	TGGGGAAGCCAAAACAGGAAG	AGCCATCAGTTTCTCTACCTCA
MyoD	CCACTCCGGGACATAGACTTG	AAAAGCGCAGGTCTGGTGAG
Myogenin	GAGACATCCCCCTATTTCTACCA	GCTCAGTCCGCTCATAGCC
Myostatin	AGTGGATCTAAATGAGGGCAGT	GTTTCCAGGCGCAGCTTAC
Pax7	TCTCCAAGATTCTGTGCCGAT	CGGGGTTCTCTCTCTTATACTCC
PDK4	AGGGAGGTCGAGCTGTTCTC	GGAGTGTTCACTAAGCGGTCA
Pgf2α synthase	CTGGACTCATCGCAAACACAA	AGGAAGCCTTTGACTTCTGTCTA
Sell	TACATTGCCCAAAAGCCCTTAT	CATCGTTCCATTTCCCAGAGTC
Sln	CTTGGGTGGATACCACGTCTG	CTTCTGTCTTACAGCCATAGCC
Sncg	AAAGACCAAGCAGGGAGTAACG	GACCACGATGTTTTCAGCCTC
Spp1	AGGAAGAAGTAGGCATTTCTGGT	TCGGCTCTGCAATGTTGTCTG
Tbc1d1	TTCCTGGGGGTGAGTCTCAG	GCAGGGCATTACGGTAGGAG
Tlr2	GCAAACGCTGTTCTGCTCAG	AGGCGTCTCCCTCTATTGTATT
Tmem26	TTCCTGTTGCATTCCCTGGTC	GCCGGAGAAAGCCATTTGT
TNF-α	CCCTCACACTCAGATCATCTTCT	GCTACGACGTGGGCTACAG
Tnnc1	GCGGTAGAACAGTTGACAGAG	CCAGCTCCTTGGTGCTGAT
Tnni1	ATGCCGGAAGTTGAGAGGAAA	TCCGAGAGGTAACGCACCTT
Tpm3	ACCACCATCGAGGCGGTAA	CCCTTTCCTCCGCATCATCA
Traf6	AAAGCGAGAGATTCTTTCCCTG	ACTGGGGACAATTCACTAGAGC
Gapdh (internal control)	AGGTCGGTGTGAACGGATTTG	TGTAGACCATGTAGTTGAGGTCA
